# Personality Traits and Cognitive Abilities in Conflict Management: Preliminary Insights from a Situational Judgment Test of Emotional Intelligence

**DOI:** 10.3390/jintelligence13110143

**Published:** 2025-11-07

**Authors:** Juliane Völker, Katja Schlegel, Marcello Mortillaro

**Affiliations:** 1 Swiss Center for Affective Sciences, University of Geneva, Chemin des Mines 9, 1202 Geneva, Switzerland; marcello.mortillaro@unige.ch; 2 Institute for Psychology, University of Bern, Fabrikstrasse 8, 3012 Bern, Switzerland; katja.schlegel@unibe.ch

**Keywords:** cognitive abilities, conflict management, personality, situational judgment test

## Abstract

Previous studies suggested that people have stable conflict management styles which relate to their personality traits. However, recent research indicates that conflict management requires flexibility to switch between strategies and that this flexibility may relate to cognitive abilities. The interplay between stable preferences and a flexible performance in conflict management is a novel research avenue. We analyzed data from four studies (*N* = 1104) using a situational judgment test on emotional intelligence that presents conflict situations in the workplace. We tested whether preferences (selecting one’s typical behavior) mainly relate to personality traits and performance (selecting the ideal behavior) to cognitive abilities. We found that preferences akin to conflict management styles emerged between individuals; however, these preferences did not reflect tangible differences in personality traits. Considering performance, cognitive abilities were consistently conducive to solving conflicts, while the contribution of personality traits varied across situations, with the possibility that some traits may even hinder conflict resolution in some situations. We provide preliminary evidence on the contributions of both traits and abilities to conflict management, arguing that conflict management research needs to adopt a holistic view that combines both a person’s traits and abilities to explain their conflict behavior.

## 1. Introduction

**Figure 1:**
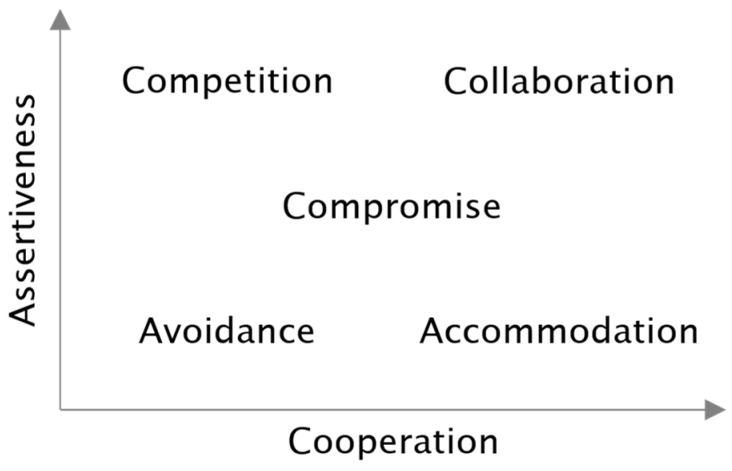
The five conflict management strategies as a function of the two dimensions of assertiveness and cooperation (Howell 2014).

Every workplace can become a stage for conflict, involving intense emotions such as anger, fear, sadness, and even inappropriate joy (Schlegel and Mortillaro 2019). To resolve such conflicts, individuals can avoid, accommodate (also called oblige, yield), compromise, compete (dominate, force), or collaborate (integrate; Rahim 1983; Rahim and Magner 1995; Thomas 1992). These five ways of responding denote distinct conflict management strategies, reflected in the two dimensions of a person’s assertiveness (their concern for their own interests) and cooperation (their concern for the interests of others; Blake and Mouton 1964). As depicted in Figure 1, competition is a strategy high in assertiveness but low in cooperation; its counterpart is accommodation, which is high in cooperation but low in assertiveness. Along the diagonal, we find both assertiveness and cooperation absent in avoidance, moderate in compromise, and high in collaboration.

People differ in their “styles”, meaning their preferences for using either of these strategies (Rahim 1983). Both researchers and practitioners are interested in understanding what determines the degrees of assertiveness or cooperation that people show during conflicts. One prevalent topic in this field is personality.

### 1.1. Understanding Preferences: The Contribution of Personality Traits

Stable preferences for using specific strategies to manage conflicts have been examined for their association with traits of the “Big Five” model of personality (Tehrani and Yamini 2020; Wood and Bell 2008). Indeed, “personality factors […] seem to play a role [in determining people’s responses to conflict], but it is not a role that is consistent […]” (Utley et al. 1989; p. 293). Three decades later, Tehrani and Yamini (2020) summarized the relationships between individuals’ use of conflict management strategies and their personality traits in a meta-analysis: those who favor strategies characterized by cooperation—namely collaboration, compromise, and accommodation—tend to be more extraverted, agreeable, conscientious, and open (0.05 < ρs < 0.36). These traits describe tendencies to show pro-social, helping, and reflective behaviors, which correspond well with seeking cooperative resolutions to conflicts. Individuals who score high in extraversion are confident in standing up for themselves, which also leads them towards more assertive behaviors (0.11 < ρs < 0.16). Finally, individuals who avoid or accommodate (low assertiveness) score higher in agreeableness (0.14 < ρs < 0.21) and neuroticism (0.05 < ρs < 0.19). Those who are compliant or anxious seem to prefer minimizing negative emotions and preserving their relationships by either yielding to the other person or by evading conflicts altogether. Echoing Utley et al.’s (1989) earlier claim, Tehrani and Yamini’s (2020) meta-analysis suggests that personality’s influence on individuals’ conflict management styles may seem reliable across studies, though not always substantial.

### 1.2. Beyond Preferences: The Contribution of Cognitive Abilities

Personality traits relate to how a person typically prefers to behave in a conflict. However, it is important to distinguish between a stable, typical preference and a specific, contextual behavior. Considering only personality as a determining factor overlooks the fact that individuals may employ strategies flexibly, considering the situational context to transform a conflict from a destructive threat into a constructive process (Howell 2014; Huan and Yazdanifard 2012). Furthermore, even though strategies such as compromise and collaboration are generally considered to be adaptive, they may not be optimal in all contexts (Speakman and Ryals 2010). In other words, a stable preference does not automatically translate into behavior nor can it be regarded as strictly adaptive or maladaptive, per se. Instead, we should expect other contextual and personal factors to influence the determination of the most appropriate strategy.

To understand whether a person can identify the most appropriate strategy and effectively engage in the corresponding behavior to manage conflicts, researchers need to look beyond stable conflict management styles and their associations with personality traits. Hence, we suggest investigating how cognitive abilities influence the capacity to adapt behavior to the demands of the present conflict situation. One candidate to start this exploration is emotional intelligence.

### 1.3. Emotional Intelligence at the Intersection of Traits and Abilities

Emotional intelligence (EI) has garnered scholarly interest in conflict and negotiation (e.g., Sharma et al. 2013), with studies reporting that individuals high in EI prefer strategies that integrate the interests of all parties—essentially, collaboration and compromise (Schlaerth et al. 2013; Shih and Susanto 2010). EI may serve as a resource that, in the heat of conflict, enables a person to regulate their own emotions, adopt the perspectives of others, and effectively negotiate a solution (Chen et al. 2019; Jordan and Troth 2004). Some studies have even found that high-EI individuals may employ a domineering strategy (competition), presumably to persuade and influence others’ feelings and opinions, for instance, in leadership contexts (Zhang et al. 2015).

While EI appears to be a promising asset for conflict management, there is a significant limitation to consider in these studies: EI is most often measured through self-report. This assessment method taps into stable self-perceptions of emotion regulation, self-esteem, and low impulsiveness (Petrides et al. 2007). This “trait-EI” approach is associated with the personality traits of emotional stability (inverse neuroticism), extraversion, openness, conscientiousness, and agreeableness (Pérez-González and Sanchez-Ruiz 2014; Petrides et al. 2007). It is therefore unsurprising that people who perceive themselves as emotionally intelligent also tend to report using constructive strategies that are both cooperative and assertive. Self-reporting methods, thus, severely limit the contribution of trait-EI over the Big Five personality traits for understanding conflict management.

However, EI can also be measured as a set of cognitive abilities related to emotional processing, such as accurate emotion perception, understanding, and the management of one’s own and others’ emotions (Mayer et al. 2016; O’Connor et al. 2019). “Ability-EI” may seem pivotal for dealing with emotions in conflict management and yet this perspective has been largely overlooked in conflict management research (Winardi et al. 2022). Some seminal studies have shown that ability-EI promotes concern for mutual interests, effective communication, and problem solving (Krishnakumar et al. 2019), and is associated with conveying an image of warmth, competence, and effectiveness in conflict management (Schlegel et al. 2025a).

Due to its conceptual duality, EI offers a suitable framework to investigate conflict management at the intersection of traits and abilities. One approach to make this duality tangible is to discern preferences or typical behavior from “maximum” performance or ideal behavior. For example, Freudenthaler and colleagues (Freudenthaler et al. 2008; Freudenthaler and Neubauer 2007) employed a situational judgment test to resolve emotional conflicts. They asked test takers “what would be the ideal thing to do” and “what they would actually do”. While responding according to the latter instruction (the typical behavior) was associated with personality traits, responding to the first instruction (the ideal behavior) was related to cognitive abilities. Mohorić et al. (2024) replicated these findings, adding that typical preferences and maximum performance correlated to moderate degrees, with high conscientiousness increasing the overlap between typical and maximum performance. Differentiation between typical and maximum performance has also been confirmed in a meta-analysis in other domains (McDaniel et al. 2007).

In the space outlined by the two dimensions of assertiveness and cooperation, an individual’s stable personality traits map their personal comfort zone, whereas behavioral flexibility to move along these dimensions may draw from cognitive resources, such as knowledge and mental capacity (Freudenthaler et al. 2008; Freudenthaler and Neubauer 2007).

### 1.4. The Present Approach

Besides these few examples, the integration of traits and abilities, typical and maximum performance and stability and flexibility, remains untapped. Adopting a dual perspective of traits and abilities towards conflict management through the lens of EI is now possible thanks to new EI measures that could be used to assess the relative contribution of the two perspectives. The present study offers a look into how personality traits and cognitive abilities contribute to conflict management in a situational judgment test. As our measure of conflict management, we utilized the emotion management subtest of the Geneva Emotional Competence Test (Schlegel and Mortillaro 2019). We considered patterns of responses to discern preferences (typical performance, akin to stable conflict management styles) from performance (maximum performance, picking the correct responses indicating flexible conflict management behavior).

## 2. Materials and Methods

### 2.1. Samples

Our present data comprises 1104 participants from four independent samples collected in previous studies (samples are labeled Hospitality, Health Care, Psychology, and General Population). Table 1 provides an overview of the sample characteristics, collected measures, and publication.

#### 2.1.1. Hospitality

The participants were German- and English-speaking students at a professional hospitality school in Switzerland. A part of this data (*N* = 330) has been published in Völker et al. (2023).

#### 2.1.2. Health Care

Participants were German-speaking students at a Swiss University of Applied Sciences, enrolled in the first semester in nursing, physiotherapy, midwifery training, or dietetics (Schlegel et al., forthcoming).

#### 2.1.3. Psychology

Participants were German-speaking undergraduate psychology students at a Swiss university who received course credit for completing the study. Aside from EI, they filled in a personality inventory and other instruments irrelevant to the present study (Xenakis et al. 2023).

#### 2.1.4. General Population

Participants were native English-speaking workers recruited through the Prolific platform (https://www.prolific.com/) who completed tests of EI, crystallized intelligence, and other measures irrelevant to the present study (Schlegel et al. 2025b).

### 2.2. Procedures

All data was collected online via the Qualtrics platform (https://www.qualtrics.com/). Approval from the ethics committees as well as informed consent was obtained from the respective institutions and participants. In each sample, the Emotion Management test was disseminated first before participants received access to a separate online survey assessing the respective remaining measures described below.

### 2.3. Measures

#### 2.3.1. Emotion Management Test (Schlegel and Mortillaro 2019)

The Emotion Management (EM) test comprises 20 items describing workplace conflicts in which another person experiences anger, fear, inappropriate joy, or sadness. For each situation, five possible reactions are presented, representing the conflict management strategies of avoidance, accommodation, competition, compromise, and collaboration (Thomas 1992). Participants select the response that best reflects how they would react in this situation (typical performance; “what do you do?”). They receive a point if they choose the behavior corresponding to the best strategy for the demands of the situation. Across the 20 situations, each of the five strategies is scored as correct four times. Hence, to achieve a high score, test takers must demonstrate flexibility to choose between different strategies. Correctness was determined using an established conflict management theory and expert validation (for a full description of these criteria, see Schlegel and Mortillaro 2019). Because of the inherent heterogeneity of test items in SJTs, test–retest coefficients are commonly considered adequate indicators of reliability (Harenbrock et al. 2023). Test–retest coefficients were not available to us; instead, internal consistency coefficients of SJTs typically range from 0.46 to 0.68 (Corstjens et al. 2017), and the EM test’s reliability was within this range (ω = 0.537).

The following example illustrates the item format (taken from Schlegel and Mortillaro 2019, p. 7): “Your colleague with whom you get along very well tells you that he is getting dismissed and that you will be taking over his projects. While he is telling you the news, he starts crying. He is very sad and desperate. You have a meeting coming up in 10 min. What do you do?” Then, a response must be selected: (1) You take some time to listen to him until you get the impression he calmed down a bit, at risk of being late for your meeting (compromise); (2) you cancel your meeting, have a coffee with him, and say that he has every right to be sad and desperate. You ask if there is anything you can do for him (accommodate); (3) you tell him that you are sorry but that your meeting is extremely important. You say that you may find some time another day this week to talk about it (avoid); (4) you suggest that he joins you for your meeting with your supervisor so that you can plan the transfer period together (collaborate); and (5) you cancel your meeting and offer him to start discussing the transfer of his projects immediately (compete). In this example, the correct response is compromise, for it addresses the colleague’s emotion while heeding the constraints of the situation.

#### 2.3.2. Ten-Item Personality Inventory (TIPI; Gosling et al. 2003)

The TIPI was used in two samples (Hospitality, Health Care). It assesses the Big Five, extraversion, agreeableness, conscientiousness, emotional stability (inverse neuroticism), and openness, with two items per trait. Reliabilities were satisfactory for conscientiousness (ω = 0.728), openness (ω = 0.733), emotional stability (ω = 0.657), and extraversion (ω = 0.640) yet unsatisfactory for agreeableness (ω = 0.141). For transparency, we report the results concerning agreeableness when measured with the TIPI; however, we interpret them cautiously.

#### 2.3.3. HEXACO Personality Inventory (Ashton and Lee 2009)

The 60-item version of the HEXACO personality inventory was employed in one sample (Psychology). It differentiates more finely along the Big Five by extracting a sixth dimension called honesty–humility (henceforth honesty) from the agreeableness dimension. Reliabilities were high for all dimensions—extraversion (ω = 0.817), conscientiousness (ω = 0.779), emotionality (corresponding to neuroticism in the Big Five model; ω = 0.772), honesty (ω = 0.738), agreeableness (ω = 0.735), and openness (ω = 0.706).

#### 2.3.4. Culture Fair Intelligence Test (CFT; Cattell and Cattell 1957)

The CFT was administered in one sample (Hospitality). It is a language-free test that assesses fluid ability. Its tasks concern the analysis of figure series, classification of figures, solving figure matrices, and inferring rules from figural presentations. The CFT contains 56 tasks. Participants were asked to solve as many as possible within 15 min. The reliability was excellent at ω = 0.909.

#### 2.3.5. Student Vocabulary Test (StuVoc1; Vermeiren et al. 2022)

The StuVoc1 measures semantic comprehension of English words as an indicator of crystallized intelligence. A 20-task version of the test was completed by one sample (General Population). In the StuVoc1, participants were presented with a test word (e.g., “period”) and an incomplete sentence that contains it (e.g., “It was a difficult <period>”). Participants must then select one out of four options, the one that correctly describes the meaning of the test word (e.g., “question,” “thing to do,” “book,” or “time”, the latter being the correct response). Reliability was satisfactory at ω = 0.829.

### 2.4. Analysis Plan

Because in the present study we aimed at assessing both preferences and performance, we analyzed responses on the EM test two-fold. To gauge preferences, we calculated the frequencies of endorsing each strategy regardless of its correctness. Following conflict management’s research tradition of determining individual conflict management styles or stable tendencies to behave (Rahim 1983; Tehrani and Yamini 2020), we performed a k-means cluster analysis based on these frequencies to group participants according to their underlying preferences for specific conflict management strategies. Subsequently, we performed Multivariate Analysis of Variance (MANOVA) to compare participants from different clusters in terms of personality traits (extraversion, agreeableness, conscientiousness, openness, emotional stability, emotionality, and honesty) and cognitive abilities (fluid ability, crystallized ability). Given earlier evidence that reports reliable associations between personality and conflict management styles (summarized by Tehrani and Yamini 2020), we hypothesized that participants from different clusters would differ in terms of personality traits but not in cognitive abilities. Additionally, we performed multiple regression analysis to determine whether personality traits and cognitive abilities predict preference scores.

To measure performance, we calculated the number of correct responses, as is the standard procedure in the EM test. Following Freudenthaler and Neubauer’s (2007) and Mohorić et al.’s (2024) examples, we used multiple regression analyses to investigate the relative contribution of personality traits and cognitive abilities in explaining performance. We hypothesized that a better performance would be positively associated with cognitive abilities but unrelated to personality traits.

## 3. Results

Analyses were run in IBM SPSS version 27. Descriptive statistics of the trait and ability measures are reported in Table 2. Descriptive statistics of the preference scores and performance scores of the EM test can be found in Table 3. Furthermore, Table A1 (Appendix A) displays the correlations between the study variables and the participants’ age and gender. Henceforth, age and gender will be treated as covariates in multivariate and regression analyses.

Section 3 is structured as follows. First, we analyzed preferences, emergent from the frequency with which participants endorse the five strategies. Second, we analyzed performance by examining the correctness of responses.

### 3.1. Preferences

#### 3.1.1. Frequency Scores

We computed frequency scores of the five conflict management strategies by adding up the times a strategy was selected. Participants selected compromise most frequently (*M* = 5.33, *SD* = 1.86), followed by collaboration (*M* = 4.77, *SD* = 2.08; *M_Diff_* to compromise = 0.556, *p* < .001), avoidance (*M* = 3.70, *SD* = 1.95; *M_Diff_* to collaboration = 1.08, *p* < .001), accommodation (*M* = 3.26, *SD* = 1.65; *M_Diff_* to avoidance = 0.438, *p* < .001), and lastly, competition (*M* = 2.94, *SD* = 1.39; *M_Diff_* to accommodation = 0.317, *p* < .001). All mean differences between scores were statistically significant, with *F*(3.61, 3978.95) = 280.28, *p* < .001, and η^2^ = 0.203 (*df*s were Greenhouse–Geisser corrected due to violation of the assumption of sphericity).

#### 3.1.2. Cluster Analysis

We z-standardized the five frequency scores and introduced them into a k-means cluster analysis to group participants with similar patterns of endorsing the conflict management strategies (Wu 2012). We explored how many clusters (*k*) best describe meaningful groups. This is achieved by testing different *k*s and observing how quickly cluster centers converge to 0. We tested a *k* of 2 to 10 with a maximum of 20 iterations to account for the possibility that participants may prefer different combinations of the five conflict management strategies. Five clusters provided the best solution, which converged after 14 iterations.

Next, we used repeated-measures MANOVA on the five frequency scores to describe the five clusters. We found that, within four clusters, participants had a strong preference for one strategy. We labeled the members of the clusters the Compromisers (*N* = 295), the Collaborators (*N* = 235), the Avoiders (*N* = 200), and the Accommodators (*N* = 157). One cluster preferred competition, compromise, and collaboration to similar degrees, whose members we labeled the Assertives (*N* = 217). An overview of the five clusters and their endorsement of strategies is provided in Table 4.

**Figure A1:**
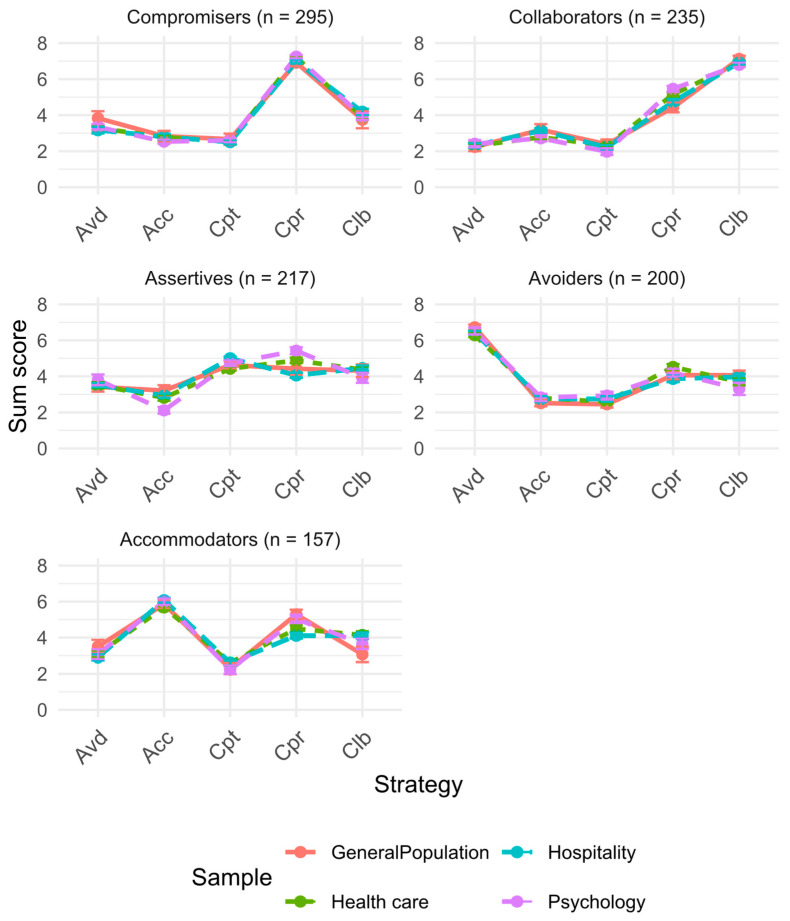
Frequency scores between the four samples depending on cluster membership. Avd = avoidance, Acc = accommodation, Cpt = competition, Cpr = compromise, and Clb = collaboration. The interaction between sample and strategy is non-significant for Compromisers, Collaborators, and Avoiders (*F*s ≤ 1.74, *p*s > .05), indicating that cluster members use all strategies with similar frequencies across all samples. The interaction is significant for Assertives (*F* = 3.59, *p* < .001) and Accommodators (*F* = 2.43, *p* = .004): cluster members from different samples vary in the frequencies with which they endorse the strategies.

Using repeated-measures MANOVA, we checked whether these patterns emerged across all four samples by introducing “sample” as a between-factor. The interaction between sample and strategy was non-significant for Compromisers, Collaborators, and Avoiders (*F*s ≤ 1.74, *p*s > .05), indicating that cluster members between the four samples did not differ in their use of strategies. The interaction was significant for Assertives (*F* = 3.59, *p* < .001) and Accommodators (*F* = 2.43, *p* = .004): Between samples, Assertives varied in their use of compromise and accommodation, and Accommodators varied in their endorsement of compromise and collaboration. These divergences do not concern the main result of the cluster analysis and are not discussed further in this manuscript. For completeness, we report additional plots of the frequency scores between samples in Figure A1 (Appendix A).

#### 3.1.3. Traits and Abilities Between Clusters

We tested whether the members of the five clusters differed in personality traits and cognitive abilities. We used multivariate MANOVA with Bonferroni-corrected pairwise comparisons in each sample separately, with age and gender as covariates, which we report in Table 5.

We expected differences in personality traits between the clusters. Contrary to our hypothesis, we found small main effects (0.027 < η^2^_p_s < 0.051) and, importantly, pairwise comparisons only approached significance (.066 < *p*s < .085). Collaborators tended to be just slightly more extraverted, agreeable, and honest than Assertives (who scored lowest in extraversion and honesty) and Avoiders (who scored lowest in agreeableness). The Compromisers scored highest in emotional stability in comparison to Avoiders (who scored the lowest). We found no significant effects concerning conscientiousness, openness, or emotionality. In terms of cognitive abilities, we found a medium effect for crystallized abilities (η^2^_p_ = 0.131); Compromisers had a higher mean score on the crystallized ability test compared to Accommodators (*p* = .005). There was no significant effect regarding fluid ability (η^2^_p_ = 0.019).

These results indicate that cognitive ability but not personality traits differed substantially between people with different preferences for conflict management strategies, leading us to reject our initial hypothesis.

#### 3.1.4. Traits and Abilities as Predictors of Preference Scores

We conducted multiple stepwise regression analyses to predict the preference scores. We entered age and gender as covariates in step 1 and personality and cognitive ability measures in step 2. Because differing personality and ability measures were used between samples, we report the results of the regression for each sample separately. Standardized regression coefficients (β), F-tests, and adjusted R^2^ are reported in Table 6.

Neither personality traits nor cognitive abilities were consistent predictors of preferences. Exceptionally, extraversion (β = 0.112, *p* = .035) contributed significantly to the collaboration preference in the Health Care sample (2%); low agreeableness (β = −0.185, *p* = .012) and low honesty (β = −0.171, *p* = .022) contributed to the preference for competition in the Psychology sample (4.4%); and a lower crystallized ability (β = −0.269, *p* = .016) contributed to the preference for accommodation in the General Population sample (6.3%).

### 3.2. Performance

#### 3.2.1. Performance Scores

The global EM score can range from 0 to 20, reflecting its 20 items. Participants solved, on average, *M* = 10.10 (*SD* = 3.02) items correctly. The five strategy scores ranged from 0 to 4, as each strategy was coded as correct in four items. Participants solved the compromise items most often (*M* = 2.30, *SD* = 1.10), followed by collaboration (*M* = 2.14, *SD* = 1.10; *M_Diff_* to compromise = 0.158, *p* < .001), avoidance (*M* = 1.99, *SD* = 1.10; *M_Diff_* to collaboration = 0.150, *p* < .001), competition (*M* = 1.92, *SD* = 0.979; *M_Diff_* to avoidance = 0.072, *p* = .081), and finally, accommodation items (*M* = 1.73, *SD* = 0.976; *M_Diff_* to competition = 0.188, *p* < .001). The mean differences between these five scores were statistically significant except for avoidance and competition, with *F*(3.88, 4284.36) = 55.63, *p* < .001, and η^2^ = 0.048 (*df*s was Greenhouse–Geisser corrected due to violation of the assumption of sphericity).

#### 3.2.2. Traits and Abilities as Predictors of Performance Scores

As with the preference scores, we conducted multiple stepwise regression analyses to predict the performance scores, with age and gender as covariates entered in step 1 and personality and cognitive ability measures in step 2. Standardized regression coefficients (β), F-tests, and adjusted R^2^ are reported in Table 7.

We hypothesized that performance, defined as the flexibility to employ different conflict management styles in accordance with the situation, would be predicted by cognitive abilities rather than personality traits. We found that, overall, personality and ability measures together predicted between 3% and 19% performance on the EM test, constituting significant explanations of variance in each of the four samples. Performance on the global EM test was predicted most strongly by crystallized ability (Global Population: β = 0.488, *p* < .001) and fluid ability (Hospitality: β = 0.284, *p* < .001), followed by honesty (Psychology: β = 0.254, *p* = .001), agreeableness (Hospitality: β = 0.147, *p* = .008), and conscientiousness (Health Care: β = 0.114, *p* = .030). The contributions of personality traits varied between samples: agreeableness and honesty attained significance in the Hospitality and Psychology sample but not in the Health Care sample. Conscientiousness explained significant portions of variance in the Health Care sample but neither in the Hospitality nor Psychology sample.

When dissecting the score into the five separate strategies, we found fluid ability to be a significant predictor for performance in all five strategies (*p*s < .05). Crystallized ability contributed to performance on items targeting strategies that are high in assertiveness, namely compromise, competition, and collaboration (*p*s < .01). Concerning personality traits, performance on avoidance items was predicted negatively by extraversion (Health Care, *p* = .021) and negatively by conscientiousness (Hospitality, *p* = .024). The performance on competition items was predicted negatively by both agreeableness and extraversion (Psychology: *p* = .042 and *p* = .046, respectively). A mix was observed for performance on accommodation items, which was predicted negatively by extraversion but positively by agreeableness (Hospitality: *p* = .025 and *p* = .049, respectively). Performance on collaboration items was predicted by agreeableness (Hospitality: *p* = .014). Finally, the performance on compromise items was predicted by agreeableness (Hospitality: *p* = .030) and honesty (Psychology: *p* = .043). Openness, emotional stability, and emotionality were not significant contributors to the EM test performance.

Our results support our hypothesis concerning that performance would be informed more by cognitive ability than personality traits, as crystallized and fluid abilities contributed consistently to performance scores. In comparison, personality traits’ contributions were smaller and varied depending on the samples and the targeted conflict management strategies.

## 4. Discussion

Research has demonstrated that personality traits contribute to stable preferences in how individuals respond to conflicts (Tehrani and Yamini 2020; Wood and Bell 2008). However, in the context of conflict resolution, it may be more adaptive to match the strategy with the circumstances rather than consistently relying on one preferred strategy; this flexibility appears to relate more to cognitive abilities than to personality traits (Howell 2014). Following this line of reasoning, we considered the duality of traits and abilities to understand the typical preferences and maximum performance in the context of emotional conflicts at work, situated in a situational judgment test framework rather than a self-report assessment. To provide preliminary data on this novel avenue, we analyzed four sets of data from the emotion management subtest of the Geneva Emotional Competence Test (Schlegel and Mortillaro 2019). This test measures the competence to respond adaptively to emotional conflicts in the workplace using the five conflict management strategies of avoidance, accommodation, competition, compromise, and collaboration.

### 4.1. Preferences: Differences in Personality Are Small

Participants’ preferences for specific conflict management strategies were evident even in a situational judgment test format. Participants formed clusters in which they preferred either compromise, collaboration, avoidance, or accommodation. Another group of participants showed a more diffuse preference for strategies generally high in assertion (competition, compromise, and collaboration). These clusters were clear across all four samples, which indicates that pronounced conflict management styles emerged across independent groups of people.

We hypothesized that cluster membership would reflect differences in personality traits. Contrary to our hypothesis, cluster members’ personality profiles were similar, although a few notable tendencies emerged: those who preferred collaboration tended to be the most extraverted, agreeable, and honest, while those favoring compromise tended to be the most emotionally stable. These results resemble earlier research summarized by Tehrani and Yamini’s (2020) meta-analysis. Personality dimensions with a strong social and emotional component (extraversion, agreeableness, honesty, and emotional stability) emerged consistently in the conflict management literature (Ayub et al. 2017). Further supporting earlier findings, we found small contributions of extraversion to collaborative preferences, as well as of agreeableness and honesty to less preference for competition. However, we did not observe any effects concerning openness and conscientiousness. Possibly, openness and conscientiousness correspond more to hypothetical reflections gathered in self-report measures, but they reflect less behavioral tendencies in a situational judgment test.

Interestingly, the only statistically significant effect of moderate size observed was that the Compromisers had a higher crystallized ability than Accommodators. Compromising involves making appeals, offers, and suggestions (Montes et al. 2012). These tactics require a certain level of eloquence, because a trade-off must be negotiated to satisfy everyone’s needs while also persuading all parties to accept losses. This skill aligns well with Compromisers’ high score in emotional stability, which may help them remain calm throughout the process of conflict resolution. Meanwhile, the Accommodators scored lowest in the vocabulary test, and this was echoed in a low crystallized ability score being predictive of a preference for accommodation: individuals with low verbal skills may neglect to voice their interests in a sufficiently assertive manner, although this assumption requires further investigation with dedicated research.

#### Contradictions to Previous Literature

The directions of some effects seem to contradict previous evidence, which appear interesting enough to address briefly despite them being statistically non-significant (.085 < *p*s < .066). Earlier research stated that people high in agreeableness favor avoidance and accommodation (Tehrani and Yamini 2020; Wood and Bell 2008). In our situational judgment test, it was those low in agreeableness who preferred avoidance. There appears to be a need to investigate agreeableness’ relation to strategies low in assertiveness (accommodation and avoidance) more deeply, as alternative explanations seem possible: do agreeable people wish to actively address a conflict via accommodation or avoidance to “be agreeable”, or is it less agreeable people who do not care enough about others’ interests to do anything else than withdraw from a conflict?

In a similar vein, earlier research found that extraverted people approach conflicts assertively (Tehrani and Yamini 2020; Wood and Bell 2008), whereas our results showed that those who scored comparatively low in extraversion favored competition. Extraverts may report standing up for their interests in self-report assessments (Espinoza et al. 2023), while in more behavioral assessments, their inclination to thrive in social relationships may encourage them to cooperate with others.

Differences between subjective self-perceptions and behavioral tendencies have not been studied in conflict management research to date, yet they seem worth exploring as they may impact workplace outcomes. For instance, researchers might investigate if either self-report or situational judgment tests are most useful to predict leadership effectiveness and competence in the context of conflict management (Schlegel et al. 2025a).

### 4.2. Performance: Cognitive Abilities Seem More Impactful than Traits

The EM score was associated with fluid and crystallized abilities, as well as agreeableness, honesty, and conscientiousness. These results mostly match those of Freudenthaler and Neubauer (2007), who reported that test takers’ typical performance was associated with openness, agreeableness, and conscientiousness, while their maximum performance was associated with cognitive abilities. Our study’s overall score correlated with abilities and traits, suggesting that the EM test with its typical performance instruction and performance scoring taps into both domains.

We employed multiple regression analyses to test whether personality and cognitive abilities contributed to performance in situations calling for different conflict management strategies. In line with our hypothesis, cognitive abilities were significant predictors of performance, as participants solved more items correctly if they had a higher fluid or crystallized ability. Cognitive abilities may contribute mental resources to flexibly switch between strategies that fit the situational demands (Toh and Yang 2024). While fluid ability predicted significant portions of variance in items targeting all five strategies, crystallized ability only contributed to strategies that involve high assertiveness. It makes sense that verbal skills are required to effectively assert oneself in conflict situations. Overall, cognitive abilities appear crucial for constructively managing conflicts (Jordan and Troth 2002, 2004; Krishnakumar et al. 2019; Morrison 2008) and deserve further attention in conflict management research (Winardi et al. 2022).

### 4.3. Context: Personality Traits as Strengths or Weaknesses?

Utley et al. (1989) emphasized that personality’s role in conflict management may be inconsistent. We found that, depending on contextual factors, personality traits may be either beneficial or detrimental in resolving conflicts.

In the sample of hospitality students, agreeableness appeared beneficial in situations calling for collaboration, compromise, or accommodation. Psychology students’ trait of honesty–humility seemed conducive to solving conflicts requiring either collaboration or compromise; however, their agreeableness seemed to hinder conflict resolution in situations requiring competition. It appears that being pro-social, thoughtful, and humble is an asset for chartering interpersonal conflicts into finding resolutions cooperatively. People high in agreeableness and honesty bring the authenticity to voice their own interests while also possessing the humility to accept alternative perspectives—and yet, in competitive situations, they may sometimes show excessive concern for the interests of others. However, we raise caution in these interpretations since one measure of agreeableness (the TIPI) had low internal consistency.

Conscientiousness and extraversion can be disadvantageous in situations where avoidance, accommodation, or competition are the most effective strategies. In some contexts, extraverts’ tendency to seek social interaction may lead them to engage in a conflict that would be better avoided; to a conscientious person, it may seem negligent to avoid a conflict, even though it may sometimes be the wisest course of action (Ayub et al. 2017). In some situations, extraverts’ desire for positivity may prevent them from engaging in competition, yet in other contexts, their assertiveness may keep them from accommodating others (Espinoza et al. 2023; Wood and Bell 2008). The inconsistent role of extraversion may strongly depend on contextual moderators (Utley et al. 1989).

The context that may be relevant in our samples is the professional orientation: Extraverted hospitality students who learn how to manage a hotel business may occasionally struggle to comply; extraverted future nurses learning to take medical responsibility for others may perceive it as wrong to avoid when, in fact, avoidance would be appropriate; and extraverted psychologists who are keen to engage with the perspectives of others may struggle to assert their own interests. The role of the work environment as a moderator between how personality may translate into effective conflict resolution is already a topical subject in leadership coaching (e.g., McCormick and Burch 2008), and it is certainly an interesting field for future applied research.

### 4.4. Limitations and Suggestions

The present paper offers a new analysis of data collected in four independent studies. The samples observed here are highly diverse, yet our findings suggest some generalizability for our results. However, we acknowledge that, to test our hypotheses, it is indispensable to conduct studies specifically designed to observe abilities and traits in conflict management.

Another weakness of our methodology is that, although the Emotion Management test was based on the conflict management framework, it still requires comparison with other conflict management measures (Schlegel and Mortillaro 2019). Our results align with similar studies (Montes et al. 2012; Reich et al. 2007; Sportsman and Hamilton 2007). However, the extent to which conflict management styles predict actual conflict behavior needs further research. This concern arises from the contradictory results we observed concerning agreeableness and extraversion and their associations with preferences for avoidance or competition. We suggest that utilizing behavioral assessments in combination with established self-report measures such as the ROCI-2 (Rahim and Magner 1995) or the DUTCH (De Dreu et al. 2001) would allow for a more holistic understanding of conflict management.

In our cluster analysis, conflict management styles seemed evident, yet we need to better understand what contributes to their emergence. Possible candidates could be organizational factors and cultural background (Kaushal and Kwantes 2006; Winardi et al. 2022), an individual’s mood and general affectivity (Montes et al. 2012) as well as motives (Niven 2016; Wood and Bell 2008), or simply a more fine-grained personality assessment (for an example, see Espinoza et al. 2023). Personality traits have many facets; hence, future studies may employ broader measures including subdimensions of traits, such as assertiveness and warmth (facets of extraversion) or compliance and trust (facets of agreeableness).

Lastly, our sample primarily comprised women (about 75%). According to some studies, men may be more inclined to utilize more competitive strategies, whereas women appear to favor cooperation (e.g., Rahim and Katz 2019). Thus, we must assume that an over-representation of women may have influenced our results.

## 5. Conclusions

Conflict management research has focused on stable styles and personality traits while utilizing self-report assessments. To uncover the contributions of cognitive abilities to conflict management, we examined response patterns in a situational judgment test of emotional conflict situations at work (Schlegel and Mortillaro 2019). Our results indicate that personality may not substantially inform conflict management styles (i.e., people’s preferences for specific strategies). Instead, the personality traits of agreeableness, honesty, conscientiousness, and extraversion may serve as small assets when conflicts are best resolved cooperatively yet may be somewhat of a hinderance in situations that require assertion. Cognitive abilities may provide mental resources and verbal eloquence to enhance flexibility and drive conflict resolution to success across various contexts.

In conclusion, we hope our preliminary results offer a first insight into the potential value of better understanding individuals’ contextualized conflict behavior as a function of both their preferences and stable traits on the one hand (self-report assessments), and their potential performance and cognitive flexibility on the other hand (situational judgment tests). Conflict management researchers need to consider more cognitive abilities, such as emotional intelligence (ability-EI), to enhance our understanding of how people manage conflicts. These insights could inform work performance, personnel training, and team coaching in a context as complex as conflict management.

## Figures and Tables

**Table 1:** Overview of the samples.

Sample	*N*	*M*_age_ (*SD*)	Gender (f, m, d)	Measures		Published in
				Traits	Abilities	
Hospitality	414	19.74 (1.58)	265, 149, 0	TIPI	CFT	Völker et al. (2023)
Health Care	392	21.89 (3.45)	344, 48, 0	TIPI		Schlegel et al. (forthcoming)
Psychology	201	22.31 (4.09)	166, 35, 1	HEXACO		Xenakis et al. (2023)
General Population	97	38.51 (11.31)	50, 47, 0		StuVoc1	Schlegel et al. (2025b)

Notes: Total *N* = 1104. See Section 2.3 for measures. Gender: f = woman, m = men, and d = diverse.

**Table 2:** Descriptive statistics of personality traits and cognitive abilities. Further statistics are provided in Table A2 (Appendix A).

Measures	Hospitality (*N* = 414)		Health Care (*N* = 392)		Psychology (*N* = 201)		General Population (*N* = 97)	
	*M*	*SD*	*M*	*SD*	*M*	*SD*	*M*	*SD*
Personality								
Extraversion ^1,2^	4.56	1.39	3.41	0.891	3.30	0.636		
Agreeableness ^1,2^	4.63	1.19	3.74	0.663	3.16	0.572		
Conscientiousness ^1,2^	5.14	1.51	3.83	0.768	3.56	0.591		
Openness ^1,2^	5.25	1.36	3.43	1.00	3.41	0.577		
Emotional stability ^1^	4.54	1.28	2.90	0.859				
Emotionality ^2^					3.39	0.616		
Honesty ^2^					3.52	0.591		
Cognitive ability								
Fluid ability ^3^	34.67	9.39						
Crystallized ability ^4^							15.21	3.82

Notes: Measures used: ^1^ TIPI used with Hospitality and Health Care samples, ^2^ HEXACO used with the Psychology sample, ^3^ CFT, and ^4^ StuVoc1. TIPI scale from 1 to 7 in the Hospitality sample, from 1 to 5 in the Health Care sample. HEXACO from 1 to 5. CFT from 0 to 56. StuVoc1 from 0 to 20.

**Table 3:** Descriptive statistics of responses on the EM test: preference scores indicating frequency of endorsing each strategy, and performance scores measuring correctness of each strategy. Further statistics are provided in Table A3 (Appendix A).

Measures	Hospitality (*N* = 414)		Health Care (*N* = 392)		Psychology (*N* = 201)		General Population (*N* = 97)	
	*M*	*SD*	*M*	*SD*	*M*	*SD*	*M*	*SD*
Preference scores								
Avoidance	3.67	1.98	3.57	1.85	3.68	1.91	4.34	2.17
Accommodation	3.51	1.83	3.10	1.47	3.00	1.56	3.32	1.58
Competition	3.22	1.59	2.76	1.13	2.76	1.34	2.87	1.38
Compromise	4.68	1.86	5.77	1.74	6.08	1.70	4.74	1.50
Collaboration	4.91	2.06	4.79	2.03	4.48	2.15	4.73	2.20
Performance scores								
Avoidance	1.65	1.05	2.19	1.10	2.16	1.01	2.30	1.09
Accommodation	1.61	1.03	1.76	0.920	1.83	0.903	1.97	1.05
Competition	1.66	1.07	2.08	0.829	2.12	0.894	1.97	1.08
Compromise	2.00	1.14	2.47	1.05	2.54	1.00	2.42	1.03
Collaboration	1.89	1.09	2.35	1.04	2.20	1.12	2.27	1.10
EM score (global)	8.81	3.30	10.86	2.47	10.86	2.48	10.93	2.93

Notes: Preference scores may range from 0 to 20, as the EM test contains 20 items. Performance scores may range from 0 to 4, as each strategy was correct in 4 items. EM (global) score may range from 0 to 20, indicating correctness on the total number of items.

**Table 4:** Repeated-measures MANOVA on mean frequency scores of the five conflict management strategies within the five clusters.

Cluster Name	*N*	Preferred Strategies		Remaining Strategies		Within-Subject Effect
		Name	*M* (*SD*)	Name	*M* (*SD*)	
Compromisers	295	Compromise ^a^	7.47 (1.06)	Collaboration ^b^ Avoidance ^c^ Accommodation ^d^ Competition ^d^	3.96 (1.48) 3.30 (1.44) 2.72 (1.15) 2.56 (0.967)	*F*(3.41, 218.06) = 121.92, *p* < .001, and η^2^ = 0.656
Collaborators	235	Collaboration ^a^	7.51 (1.34)	Compromise ^b^ Accommodation ^c^ Avoidance ^d^ Competition ^d^	5.01 (1.33) 2.93 (1.23) 2.31 (1.21) 2.23 (1.06)	*F*(4, 324) = 197.83, *p* < .001, and η^2^ = 0.710
Assertives	217	Competition ^a^ Compromise ^ab^ Collaboration ^b^	4.79 (0.927) 4.46 (1.45) 4.38 (1.60)	Avoidance ^c^ Accommodation ^d^	3.54 (1.37) 2.83 (1.22)	*F*(3.35, 392.18) = 31.58, *p* < .001, and η^2^ = 0.213
Avoiders	200	Avoidance ^a^	6.60 (1.17)	Compromise ^b^ Collaboration ^b^ Accommodation ^c^ Competition ^c^	4.16 (1.42) 3.83 (1.61) 2.74 (1.27) 2.68 (1.13)	*F*(3.64, 269.19) = 72.84, *p* < .001, and η^2^ = 0.496
Accommodators	157	Accommodation ^a^	6.03 (1.15)	Compromise ^b^ Collaboration ^c^ Avoidance ^d^ Competition ^e^	4.47 (1.36) 3.95 (1.53) 3.04 (1.39) 2.52 (1.16)	*F*(4, 292) = 62.47, *p* < .001, and η^2^ = 0.461

Notes: *N* = 1104. The *df*s were Greenhouse–Geisser corrected for Compromisers, Assertives, and Avoiders due to violation of the assumption of sphericity. Superscript letters denote pairwise comparisons: same letters indicate no differences (*p*s > .05); different letters indicate significant differences (*p*s < .05). Women (*N* = 825) and men (*N* = 279) were distributed evenly across the five clusters, χ^2^(4) = 1.30, and *p* = .861.

**Table 5:** Multivariate MANOVA main effects between members of the five clusters on personality and ability measures.

Measures	Hospitality (*N* = 414)			Health Care (*N* = 392)			Psychology (*N* = 201)			General Population (*N* = 97)		
	*F*(4, 407)	*p*	η^2^_p_	*F*(4, 385)	*p*	η^2^_p_	*F*(4, 193)	*p*	η^2^_p_	*F*(4, 90)	*p*	η^2^_p_
Personality												
Extraversion ^1,2^	2.28	.060	0.022	2.65	.033 *	0.027	2.16	.076	0.043			
Agreeableness ^1,2^	0.296	.881	0.003	1.40	.232	0.014	2.54	.041 *	0.050			
Conscientiousness ^1,2^	0.722	.578	0.007	1.55	.187	0.016	0.586	.673	0.012			
Openness ^1,2^	0.609	.656	0.006	1.02	.396	0.011	0.565	.689	0.012			
Emotional stability ^1^	0.505	.732	0.005	2.80	.026 *	0.028						
Emotionality ^2^							0.324	.862	0.007			
Honesty ^2^							2.59	.038 *	0.051			
Cognitive ability												
Fluid ability ^3^	1.94	.103	0.019									
Crystallized ability ^4^										3.39	.012 *	0.131
Post hoc test				Extraversion: Collaborators > Assertives, *M_Diff_* = 0.415, and *p* = .066 Emotional Stability: Compromisers > Avoiders, *M_Diff_* = 0.331, and *p* = .085			Agreeableness: Collaborators > Avoiders, *M_Diff_* = 0.381, and *p* = .066 Honesty: Collaborators > Assertives, *M_Diff_* = 0.382, and *p* = .084			Crystallized ability: Compromisers > Accommodators, *M_Diff_* = 0.237, and *p* = .005 **		

Notes: Assessed via ^1^ TIPI, ^2^ HEXACO, ^3^ CFT, and ^4^ StuVoc1. We used Bonferroni adjustment on post hoc tests to test pairwise comparisons between the five levels of cluster membership. Age and gender were introduced as covariates. * *p* < .05, ** *p* < .01.

**Table 6:** Multiple regression of personality and ability measures on EM preference scores for the four samples.

Hospitality Sample (*N* = 414)					
Predictors	Strategy				
	Avoidance	Accommodation	Competition	Compromise	Collaboration
	β	β	β	β	β
Covariates					
Age	0.113 *	−0.051	0.050	−0.131 **	0.017
Gender	−0.036	−0.058	−0.051	0.139 **	0.000
Personality					
Extraversion ^1,2^	0.005	−0.092	−0.073	0.020	0.114
Agreeableness ^1,2^	−0.068	−0.015	−0.024	0.051	0.050
Conscientiousness ^1,2^	−0.062	0.086	−0.065	0.002	0.031
Openness ^1,2^	−0.018	0.091	−0.007	−0.039	−0.022
Emotional stability ^1^	0.046	−0.064	−0.026	0.071	−0.032
Emotionality ^2^					
Honesty ^2^					
Cognitive ability					
Fluid ability ^3^	−0.034	−0.085	−0.096	0.085	0.105 *
Crystallized ability ^4^					
*F*(8, 405)	*F* = 1.44, *p* = .179	*F* = 1.77, *p* = .081	*F* = 1.66, *p* = .107	*F* = 2.48, *p* = .012	*F* = 1.43, *p* = .182
*R*^2^ (adj)	0.008	0.015	0.013	0.028	0.008
Health Care sample (*N* = 392)					
Predictors	Strategy				
	Avoidance	Accommodation	Competition	Compromise	Collaboration
	β	β	β	β	β
Covariates					
Age	−0.024	−0.063	−0.112 *	0.006	0.125 *
Gender	0.007	0.118 *	0.041	−0.081	−0.045
Personality					
Extraversion ^1,2^	−0.151 **	0.041	−0.031	0.016	0.112 *
Agreeableness ^1,2^	−0.054	0.000	−0.130 *	0.028	0.098
Conscientiousness ^1,2^	0.004	−0.064	−0.014	0.047	0.010
Openness ^1,2^	0.033	0.054	0.018	−0.077	−0.013
Emotional stability ^1^	0.029	−0.061	0.008	0.111 *	−0.082
Emotionality ^2^					
Honesty ^2^					
Cognitive ability					
Fluid ability ^3^					
Crystallized ability ^4^					
*F*(7, 384)	*F* = 1.39, *p* = .209	*F* = 1.43, *p* = .190	*F* = 1.68, *p* = .113	*F* = 1.62, *p* = .127	*F* = 2.15, *p* = .038
*R*^2^ (adj)	0.007	0.008	0.012	0.011	0.020 *
Psychology sample (*N* = 201)					
Predictors	Strategy				
	Avoidance	Accommodation	Competition	Compromise	Collaboration
	β	β	β	β	β
Covariates					
Age	0.044	−0.013	−0.022	0.019	−0.031
Gender	−0.022	−0.224 **	−0.032	0.197 *	0.047
Personality					
Extraversion ^1,2^	−0.153 *	0.022	−0.090	0.125	0.077
Agreeableness ^1,2^	−0.085	0.066	−0.185 *	0.064	0.093
Conscientiousness ^1,2^	0.114	−0.096	0.054	−0.081	−0.002
Openness ^1,2^	−0.073	−0.116	0.030	0.009	0.124
Emotional stability ^1^					
Emotionality ^2^	−0.156	0.021	−0.019	0.205 *	−0.026
Honesty ^2^	−0.054	−0.013	−0.171 *	0.032	0.139
Cognitive ability					
Fluid ability ^3^					
Crystallized ability ^4^					
*F*(8, 191)	*F* = 1.58, *p* = .132	*F* = 1.65, *p* = .112	*F* = 2.15, *p* = .033	*F* = 1.50, *p* = .159	*F* = 1.51, *p* = .154
*R*^2^ (adj)	0.023	0.026	0.044 *	0.020	0.020
General Population sample (*N* = 97)					
Predictors	Strategy				
	Avoidance	Accommodation	Competition	Compromise	Collaboration
	β	β	β	β	β
Covariates					
Age	−0.117	−0.210	0.017	−0.056	−0.239 *
Gender	−0.114	−0.203 *	0.062	−0.113	−0.165
Personality					
Extraversion ^1,2^					
Agreeableness ^1,2^					
Conscientiousness ^1,2^					
Openness ^1,2^					
Emotional stability ^1^					
Emotionality ^2^					
Honesty ^2^					
Cognitive ability					
Fluid ability ^3^					
Crystallized ability ^4^	0.051	0.198	0.360 **	0.383 **	0.350 **
*F*(3, 93)	*F* = 0.634, *p* = .595	*F* = 2.51, *p* = .064	*F* = 5.03, *p* = .003	*F* = 5.07, *p* = .003	*F* = 4.27, *p* = .007
*R*^2^ (adj)	−0.012	0.045	0.112 **	0.113 **	0.093 **

Notes: Regression method: Enter. Step 1: covariates, step 2: personality and ability measures. Only step 2 is shown. Measures used: ^1^ TIPI, ^2^ HEXACO, ^3^ CFT, and ^4^ StuVoc1. Fields remain blank if the measure was not collected in the respective sample. Gender: 1 = women, 2 = men. * *p* < .05, and ** *p* < .01.

**Table 7:** Multiple regression of personality and ability measures on EM performance scores for the four samples.

Hospitality Sample (*N* = 414)						
Predictors	EM (global)	Strategy				
		Avoidance	Accommodation	Competition	Compromise	Collaboration
	β	β	β	β	β	β
Covariates						
Age	0.001	0.029	−0.027	0.052	−0.113 *	0.067
Gender	−0.084	−0.152 **	−0.079	−0.009	0.000	−0.024
Personality						
Extraversion ^1,2^	−0.043	−0.036	−0.129 *	−0.041	0.027	0.038
Agreeableness ^1,2^	0.147 **	0.037	0.112 *	0.039	0.121 *	0.138 *
Conscientiousness ^1,2^	−0.007	−0.139 *	0.082	−0.107	0.057	0.082
Openness ^1,2^	0.020	0.014	0.063	−0.016	−0.013	0.017
Emotional stability ^1^	−0.036	0.018	−0.072	−0.047	0.060	−0.072
Emotionality ^2^						
Honesty ^2^						
Cognitive ability						
Fluid ability ^3^	0.284 ***	0.129 **	0.115 *	0.175 ***	0.233 ***	0.209 ***
Crystallized ability ^4^						
*F*(8, 405)	*F* = 6.29, *p* < .001	*F* = 3.11, *p* = .002	*F* = 3.23, *p* = .001	*F* = 2.93, *p* = .003	*F* = 5.80, *p* < .001	*F* = 4.72, *p* < .001
*R*^2^ (adj)	0.093 **	0.039 **	0.041 **	0.036 **	0.085 **	0.067 **
Health Care sample (*N* = 392)						
Predictors	EM (global)	Strategy				
		Avoidance	Accommodation	Competition	Compromise	Collaboration
	β	β	β	β	β	β
Covariates						
Age	0.103 *	0.066	−0.025	0.039	0.068	0.096
Gender	−0.124 *	−0.087	0.078	−0.021	−0.125 *	−0.128 *
Personality						
Extraversion ^1,2^	−0.071	−0.123 *	−0.053	−0.021	−0.057	0.082
Agreeableness ^1,2^	−0.043	−0.073	−0.059	−0.091	0.053	0.044
Conscientiousness ^1,2^	0.114 *	0.076	0.089	0.001	0.059	0.050
Openness ^1,2^	0.094	0.043	0.098	0.095	−0.016	0.032
Emotional stability ^1^	−0.038	0.028	−0.065	0.017	0.018	−0.094
Emotionality ^2^						
Honesty ^2^						
Cognitive ability						
Fluid ability ^3^						
Crystallized ability ^4^						
*F*(7, 384)	*F* = 2.65, *p* = .011	*F* = 1.82, *p* = .103	*F* = 1.47, *p* = .176	*F* = 1.05, *p* = .398	*F* = 1.49, *p* = .168	*F* = 2.48, *p* = .017
*R*^2^ (adj)	0.029 *	0.013	0.008	0.001	0.009	0.026 *
Psychology sample (*N* = 201)						
Predictors	EM (global)	Strategy				
		Avoidance	Accommodation	Competition	Compromise	Collaboration
	β	β	β	β	β	β
Covariates						
Age	0.149 *	0.102	−0.025	0.081	0.168 *	0.041
Gender	0.015	0.034	−0.163	0.001	0.090	0.052
Personality						
Extraversion ^1,2^	−0.113	−0.072	−0.108	−0.144 *	−0.015	0.030
Agreeableness ^1,2^	0.027	0.008	−0.021	−0.152 *	0.134	0.072
Conscientiousness ^1,2^	0.056	0.100	0.021	0.068	−0.051	0.009
Openness ^1,2^	−0.042	−0.088	−0.143	0.043	−0.064	0.124
Emotional stability ^1^						
Emotionality ^2^	−0.003	−0.034	−0.060	−0.082	0.155	0.000
Honesty ^2^	0.254 **	0.129	0.086	−0.043	0.150 *	0.276 ***
Cognitive ability						
Fluid ability ^3^						
Crystallized ability ^4^						
*F*(8, 191)	*F* = 3.02, *p* = .003	*F* = 1.30, *p* = .247	*F* = 1.67, *p* = .108	*F* = 1.33, *p* = .232	*F* = 2.21, *p* = .028	*F* = 3.21, *p* = .002
*R*^2^ (adj)	0.075 **	0.012	0.026	0.013	0.046 *	0.081 **
General Population sample (*N* = 97)						
Predictors	EM (global)	Strategy				
		Avoidance	Accommodation	Competition	Compromise	Collaboration
	β	β	β	β	β	β
Covariates						
Age	−0.221 *	−0.117	−0.210	0.017	−0.056	−0.239 *
Gender	−0.193 *	−0.114	−0.203 *	0.062	−0.113	−0.165
Personality						
Extraversion ^1,2^						
Agreeableness ^1,2^						
Conscientiousness ^1,2^						
Openness ^1,2^						
Emotional stability ^1^						
Emotionality ^2^						
Honesty ^2^						
Cognitive ability						
Fluid ability ^3^						
Crystallized ability ^4^	0.488 ***	0.051	0.198	0.360 **	0.383 **	0.350 **
*F*(3, 93)	*F* = 8.46, *p* < .001	*F* = 0.634, *p* = .595	*F* = 2.51, *p* = .064	*F* = 5.03, *p* = .003	*F* = 5.07, *p* = .003	*F* = 4.27, *p* = .007
*R*^2^ (adj)	0.189 ***	−0.012	0.045	0.112 **	0.113 **	0.093 **

Notes: Regression method: Enter. Step 1: covariates, step 2: personality and ability measures. Only step 2 is shown. Measures used: ^1^ TIPI, ^2^ HEXACO, ^3^ CFT, and ^4^ StuVoc1. Fields remain blank if the measure was not collected in the respective sample. Gender: 1 = women, 2 = men. * *p* < .05, ** *p* < .01, and *** *p* < .001.

## Data Availability

The original data presented in the study are openly available in Open Science Framework (OSF) at https://osf.io/2abzh/overview?view_only=7212c1d001db42fdbb05c8e387eed08f (7 September 2025).

## Abbreviations

The following abbreviations are used in this manuscript:

TIPI	Ten-item personality inventory (Gosling et al. 2003)
HEXACO	HEXACO (honesty-humility, emotionality, extraversion, agreeableness, conscientiousness, openness) personality inventory (Ashton and Lee 2009)
CFT	Culture fair intelligence test (Cattell and Cattell 1957)
StuVoc1	Student vocabulary test (Vermeiren et al. 2022)

## Appendix A

**Table A1:** Correlation analysis between study variables and participants’ age and gender.

Measures	Age		Gender	
	*r*	*p*	*r*	*p*
Preference scores				
Avoidance	0.079	.008 **	0.026	.386
Accommodation	−0.053	.080	0.005	.876
Competition	−0.033	.271	0.033	.270
Compromise	−0.022	.466	−0.046	.129
Collaboration	0.009	.757	−0.010	.750
Performance scores				
Avoidance	0.109	<.001 ***	−0.120	<.001 ***
Accommodation	0.040	.183	−0.078	.009 **
Competition	0.092	.002 **	−0.039	.196
Compromise	0.089	.003 **	−0.089090	.003 **
Collaboration	0.069	.021 *	−0.105	<.001 ***
EM score (global)	0.140	<.001 ***	−0.152	<.001 ***
Personality (TIPI)				
Extraversion	−0.160	<.001 ***	0.081	.021 *
Agreeableness	−0.189	<.001 ***	0.060	.088
Conscientiousness	−0.238	<.001 ***	0.080	.023 *
Openness	−0.208	<.001 ***	0.125	<.001 ***
Emotional stability	−0.159	<.001 ***	0.252	<.001 ***
Personality (HEXACO)				
Extraversion	0.113	.111	0.049	.488
Agreeableness	−0.002	.974	0.129	.067
Conscientiousness	−0.071	.316	−0.148	.037 *
Openness	0.122	.087	−0.136	.055
Emotionality	−0.193	.006 **	−0.493	<.001 ***
Honesty	0.148	.037 *	−0.190	.007 **
Cognitive ability				
Fluid ability	0.003	.949	−0.044	.375
Crystallized ability	0.436	<.001 ***	0.018	.861

Notes: Correlation coefficient = Pearson’s *r*. Gender was coded as 1 = women, 2 = men. * *p* < .05, ** *p* < .01, and *** *p* < .001.

**Table A2:** Further descriptive statistics of personality traits and cognitive abilities.

Measures	Hospitality (*N* = 414)			Health Care (*N* = 392)			Psychology (*N* = 201)			General Population (*N* = 97)		
	*Min*	*Max*	*Skew*	*Min*	*Max*	*Skew*	*Min*	*Max*	*Skew*	*Min*	*Max*	*Skew*
Personality												
Extraversion ^1,2^	1.00	7.00	−0.247	1.00	5.00	−0.222	1.60	4.60	−0.276			
Agreeableness ^1,2^	1.00	7.00	−0.489	1.50	5.00	−0.521	1.38	4.25	−0.403			
Conscientiousness ^1,2^	1.00	7.00	−1.03	1.00	5.00	−0.471	2.10	4.80	−0.262			
Openness ^1,2^	1.00	7.00	−1.10	1.00	5.00	−0.138	1.70	4.60	−0.262			
Emotional stability ^1^	1.00	7.00	−0.245	1.00	5.00	−0.110						
Emotionality ^2^							1.80	4.70	−0.144			
Honesty ^2^							1.90	5.00	−0.214			
Cognitive ability												
Fluid ability ^3^	0.00	52.00	−1.36									
Crystallized ability ^4^										4.00	20.00	−1.03

Notes: Skew = skewness. Measures used: ^1^ TIPI used with Hospitality and Health Care samples, ^2^ HEXACO used with the Psychology sample, ^3^ CFT, and ^4^ StuVoc1. TIPI scale from 1 to 7 in the Hospitality sample, from 1 to 5 in the Health Care sample. HEXACO from 1 to 5. CFT from 0 to 56. StuVoc1 from 0 to 20.

**Table A3:** Further descriptive statistics of responses on the EM test: preference scores indicating frequency of endorsing each strategy and performance scores measuring correctness of each strategy.

Measures	Hospitality (*N* = 414)			Health Care (*N* = 392)			Psychology (*N* = 201)			General Population (*N* = 97)		
	*Min*	*Max*	*Skew*	*Min*	*Max*	*Skew*	*Min*	*Max*	*Skew*	*Min*	*Max*	*Skew*
Preference scores												
Avoidance	0.00	13.00	0.550	0.00	11.00	0.468	0.00	10.00	0.501	0.00	10.00	0.240
Accommodation	0.00	9.00	0.553	0.00	8.00	466	0.00	8.00	0.800	0.00	7.00	0.478
Competition	0.00	8.00	0.253	0.00	7.00	0.216	0.00	7.00	0.277	0.00	7.00	0.221
Compromise	0.00	11.00	0.166	0.00	11.00	0.061	1.00	11.00	0.085	1.00	8.00	−0.196
Collaboration	0.00	12.00	0.415	0.00	12.00	0.353	0.00	10.00	0.166	0.00	11.00	0.415
Performance scores												
Avoidance	0.00	4.00	0.126	0.00	4.00	−0.060	0.00	4.00	−0.003	0.00	4.00	−0.230
Accommodation	0.00	4.00	0.112	0.00	4.00	−0.106	0.00	4.00	0.063	0.00	4.00	0.063
Competition	0.00	4.00	0.293	0.00	4.00	−0.122	0.00	4.00	−0.290	0.00	4.00	0.062
Compromise	0.00	4.00	−0.050	0.00	4.00	−0.344	0.00	4.00	−0.315	0.00	4.00	−0.403
Collaboration	0.00	4.00	0.081	0.00	4.00	−0.275	0.00	4.00	−0.195	0.00	4.00	−0.117
EM score (global)	0.00	17.00	−0.004	2.00	17.00	−0.415	3.00	16.00	−0.587	4.00	18.00	−0.307

Notes: Skew = skewness. Preference scores may range from 0 to 20, as the EM test contains 20 items. Performance scores may range from 0 to 4, as each strategy was correct in 4 items. EM (global) score may range from 0 to 20, indicating correctness on the total number of items.

## References

[B1-jintelligence-13-00143] Ashton Michael C., Lee Kibeom (2009). The HEXACO-60: A Short Measure of the Major Dimensions of Personality. Journal of Personality Assessment.

[B2-jintelligence-13-00143] Ayub Nailah, AlQurashi Susan M., Al-Yafi Wafa A., Jehn Karen (2017). Personality traits and conflict management styles in predicting job performance and conflict. International Journal of Conflict Management.

[B3-jintelligence-13-00143] Blake Robert R., Mouton Jane S. (1964). The Managerial Grid: The Key to Leadership Excellence.

[B4-jintelligence-13-00143] Cattell Raymond B., Cattell Alberta Karen S. (1957). Test of “g”: Culture Fair.

[B5-jintelligence-13-00143] Chen Helen X., Xu Xuemei, Phillips Patrick (2019). Emotional intelligence and conflict management styles. International Journal of Organizational Analysis.

[B6-jintelligence-13-00143] Corstjens Jan, Lievens Filip, Krumm Stefan (2017). Situational Judgement Tests for Selection. The Wiley Blackwell Handbook of the Psychology of Recruitment, Selection and Employee Retention.

[B7-jintelligence-13-00143] De Dreu Carsten K. W., Evers Arne, Beersma Bianca, Kluwer Esther S., Nauta Aukje (2001). A theory-based measure of conflict management strategies in the workplace. Journal of Organizational Behavior.

[B8-jintelligence-13-00143] Espinoza Jose A., O’Neill Thomas A., Donia Magda B. L. (2023). Big Five factor and facet personality determinants of conflict management styles. Personality and Individual Differences.

[B9-jintelligence-13-00143] Freudenthaler Heribert Harald, Neubauer Aljoscha C., Haller Ursula (2008). Emotional Intelligence: Instruction effects and sex differences in emotional management abilities. Journal of Individual Differences.

[B10-jintelligence-13-00143] Freudenthaler Heribert Harald, Neubauer Aljoscha C. (2007). Measuring emotional management abilities: Further evidence of the importance to distinguish between typical and maximum performance. Personality and Individual Differences.

[B11-jintelligence-13-00143] Gosling Samuel D., Rentfrow Peter J., Swann William B. (2003). A very brief measure of the Big-Five personality domains. Journal of Research in Personality.

[B12-jintelligence-13-00143] Harenbrock Jana, Forthmann Boris, Holling Heinz (2023). Retest Reliability of Situational Judgment Tests. Journal of Personnel Psychology.

[B13-jintelligence-13-00143] Howell Sally Erin (2014). Conflict Management: A Literature Review and Study. Radiology Management 14–20.

[B14-jintelligence-13-00143] Huan, Jin Lim, Yazdanifard Rashad (2012). The Difference of Conflict Management Styles and Conflict Resolution in Workplace. Business & Entrepreneurship Journal.

[B15-jintelligence-13-00143] Jordan Peter J., Troth Ashlea C. (2002). Emotional Intelligence and Conflict Resolution: Implications for Human Resource Development. Advances in Developing Human Resources.

[B16-jintelligence-13-00143] Jordan Peter J., Troth Ashlea C. (2004). Managing emotions during team problem solving: Emotional intelligence and conflict resolution. Human Performance.

[B17-jintelligence-13-00143] Kaushal Ritu, Kwantes Catherine T. (2006). The role of culture and personality in choice of conflict management strategy. International Journal of Intercultural Relations.

[B18-jintelligence-13-00143] Krishnakumar Sukumarakurup, Perera Buddhika, Hopkins Kay, Robinson Michael D. (2019). On being nice and effective: Work-related emotional intelligence and its role in conflict resolution and interpersonal problem-solving. Conflict Resolution Quarterly.

[B19-jintelligence-13-00143] Mayer John D., Caruso David R., Salovey Peter (2016). The Ability Model of Emotional Intelligence: Principles and Updates. Emotion Review.

[B20-jintelligence-13-00143] McCormick Ian, Burch Giles S. J. (2008). Personality-focused coaching for leadership development. Consulting Psychology Journal: Practice and Research.

[B21-jintelligence-13-00143] McDaniel Michael A., Hartman Nathan S., Whetzel Deborah L., Grubb W. Lee (2007). Situational judgment tests, response instructions, and validity: A meta-analysis. Personnel Psychology.

[B22-jintelligence-13-00143] Mohorić Tamara, Takšić Vladimir, Pilepić Ana Ćosić, Brown Luke E. R., Birney Damian P., MacCann Carolyn (2024). Similarities and differences between typical- and maximum-performance in emotion management situational judgment tests. Personality and Individual Differences.

[B23-jintelligence-13-00143] Montes Carlos, Rodríguez Dámaso, Serrano Gonzalo (2012). Affective choice of conflict management styles. International Journal of Conflict Management.

[B24-jintelligence-13-00143] Morrison Jeanne (2008). The relationship between emotional intelligence competencies and preferred conflict-handling styles. Journal of Nursing Management.

[B25-jintelligence-13-00143] Niven Karen (2016). Why do people engage in interpersonal emotion regulation at work?. Organizational Psychology Review.

[B26-jintelligence-13-00143] O’Connor Peter J., Hill Andrew, Kaya Maria, Martin Brett (2019). The measurement of emotional intelligence: A critical review of the literature and recommendations for researchers and practitioners. Frontiers in Psychology.

[B27-jintelligence-13-00143] Petrides Konstantinos V., Pita Ria, Kokkinaki Flora (2007). The location of trait emotional intelligence in personality factor space. British Journal of Psychology.

[B28-jintelligence-13-00143] Pérez-González Juan-Carlos, Sanchez-Ruiz Maria-Jose (2014). Trait emotional intelligence anchored within the Big Five, Big Two and Big One frameworks. Personality and Individual Differences.

[B29-jintelligence-13-00143] Rahim M. Afzalur (1983). A Measure of Styles of Handling Interpersonal Conflict. Academy of Management Journal.

[B30-jintelligence-13-00143] Rahim M. Afzalur, Katz Jeffrey P. (2019). Forty years of conflict: The effects of gender and generation on conflict-management strategies. International Journal of Conflict Management.

[B31-jintelligence-13-00143] Rahim M. Afzalur, Magner Nace R. (1995). Confirmatory factor analysis of the styles of handling interpersonal conflict: First-order factor model and its invariance across groups. Journal of Applied Psychology.

[B32-jintelligence-13-00143] Reich Warren A., Wagner-Westbrook Bonnie J., Kressel Kenneth (2007). Actual and Ideal Conflict Styles and Job Distress in a Health Care Organization. The Journal of Psychology.

[B33-jintelligence-13-00143] Schlaerth Andrea, Ensari Nurcan, Christian Julie (2013). A meta-analytical review of the relationship between emotional intelligence and leaders’ constructive conflict management. Group Processes and Intergroup Relations.

[B34-jintelligence-13-00143] Schlegel Katja, Mortillaro Marcello (2019). The Geneva Emotional Competence Test (GECo): An ability measure of workplace emotional intelligence. Journal of Applied Psychology.

[B35-jintelligence-13-00143] Schlegel Katja, de Jong Monica, Boros Smaranda (2025a). Conflict management 101: How emotional intelligence can make or break a manager. International Journal of Conflict Management.

[B36-jintelligence-13-00143] Schlegel Katja, Sommer Nils R., Heise Maja Forthcoming. Emotional intelligence and academic performance in the field of healthcare. Manuscript in preparation.

[B37-jintelligence-13-00143] Schlegel Katja, Sommer Nils R., Mortillaro Marcello (2025b). Large language models are proficient in solving and creating emotional intelligence tests. Communications Psychology.

[B38-jintelligence-13-00143] Sharma Sudeep, Bottom William P., Elfenbein Hillary Anger (2013). On the role of personality, cognitive ability, and emotional intelligence in predicting negotiation outcomes: A meta-analysis. Organizational Psychology Review.

[B39-jintelligence-13-00143] Shih Hsi-An, Susanto Ely (2010). Conflict management styles, emotional intelligence, and job performance in public organizations. International Journal of Conflict Management.

[B40-jintelligence-13-00143] Speakman James, Ryals Lynette (2010). A re-evaluation of conflict theory for the management of multiple, simultaneous conflict episodes. International Journal of Conflict Management.

[B41-jintelligence-13-00143] Sportsman Susan, Hamilton Patti (2007). Conflict Management Styles in the Health Professions. Journal of Professional Nursing.

[B42-jintelligence-13-00143] Tehrani Hossein Dabiriyan, Yamini Sara (2020). Personality traits and conflict resolution styles: A meta-analysis. Personality and Individual Differences.

[B43-jintelligence-13-00143] Thomas Kenneth W. (1992). Conflict and conflict management: Reflections and update. Journal of Organizational Behavior.

[B44-jintelligence-13-00143] Toh Wai Xing, Yang Hwajin (2024). To switch or not to switch? Individual differences in executive function and emotion regulation flexibility. Emotion.

[B45-jintelligence-13-00143] Utley Mary E., Richardson Deborah R., Pilkington Constance J. (1989). Personality and interpersonal conflict management. Personality and Individual Differences.

[B46-jintelligence-13-00143] Vermeiren Hanke, Vandendaele Aaron, Brysbaert Marc (2022). Validated tests for language research with university students whose native language is English: Tests of vocabulary, general knowledge, author recognition, and reading comprehension. Behavior Research Methods.

[B47-jintelligence-13-00143] Völker Juliane, Blal Inès, Mortillaro Marcello (2023). Emotional intelligence matters in hospitality education: Contributions of emotional intelligence, fluid ability, and personality to hospitality grades. Frontiers in Psychology.

[B48-jintelligence-13-00143] Winardi Michael Aswin, Prentice Catherine, Weaven Scott (2022). Systematic literature review on emotional intelligence and conflict management. Journal of Global Scholars of Marketing Science.

[B49-jintelligence-13-00143] Wood Valerie Ford, Bell Paul A. (2008). Predicting interpersonal conflict resolution styles from personality characteristics. Personality and Individual Differences.

[B50-jintelligence-13-00143] Wu Junjie (2012). Cluster Analysis and K-means Clustering: An Introduction. Advances in K-Means Clustering: A Data Mining Thinking.

[B51-jintelligence-13-00143] Xenakis Dimitris, Samikwa Eric, Ajayi Jesutofunmi, Di Maio Antonio, Braun Torsten, Schlegel Katja (2023). Towards Personality Detection and Prediction using Smartphone Sensor Data. Paper presented at 2023 21st Mediterranean Communication and Computer Networking Conference (MedComNet).

[B52-jintelligence-13-00143] Zhang Su Juan, Chen Yong Qiang, Sun Hui (2015). Emotional intelligence, conflict management styles, and innovation performance: An empirical study of Chinese employees. International Journal of Conflict Management.

